# Factors Contributing to CO Uptake and Elimination in the Body: A Critical Review

**DOI:** 10.3390/ijerph17020528

**Published:** 2020-01-14

**Authors:** Ke-Ting Pan, Giovanni S. Leonardi, Ben Croxford

**Affiliations:** 1 Bartlett School of Environment, Energy and Resources, University College London, London WC1E 6BT, UK; pankt930@gmail.com; 2 Graduate Institute of Aerospace and Undersea Medicine, National Defence Medical Centre, Taipei 114, Taiwan; 3 Centre for Radiation, Chemical and Environmental Hazards, Public Health England, Didcot OX11 0RQ, UK; Giovanni.Leonardi@phe.gov.uk; 4 Department of Public Health, Environments and Society, London School of Hygiene and Tropical Medicine, London WC1H 9SH, UK

**Keywords:** carbon monoxide, CO uptake, CO elimination

## Abstract

Background: Carbon monoxide (CO) poisoning is an important public health issue around the world. Research indicates that many factors may be related to the rate of CO uptake and elimination in the human body. However, some factors related to CO uptake and elimination are considered controversial. Relatively little attention has been devoted to review and synthesis of factors affecting CO uptake and elimination. Purpose: This paper provides a critical scoping review of the factors and divides them into four aspects, including environmental, demographic, physiological and treatment factors. Methods: We searched the scientific databases for research that has proposed a mathematical equation as a synthesis of quantities related to CO poisoning, CO elimination, CO uptake, CO half-life, CO uptake and elimination and their relationships. After excluding the studies that did not meet the study criteria, there were 39 studies included in the review and the search was completed before 16 December 2019. Results and conclusion: This review discusses most of the factors that impact the rate of CO uptake and elimination. Several factors may be related to CO uptake and elimination, such as CO concentration, the duration of exposure to CO, age, sex, exercise, minute ventilation, alveolar ventilation, total haemoglobin mass and different treatments for CO poisoning. Although some potential factors were not included in the review, the findings are useful by presenting an overview for discussing factors affecting CO uptake and elimination and provide a starting point for further study regarding strategies for CO poisoning and the environmental standard of CO.

## 1. Introduction

Exogenous carbon monoxide (CO) results from the incomplete combustion of carbon-containing molecules, and endogenous CO is formed within the body by metabolic processes [1]. CO is a neurotransmitter in the brain and peripheral autonomic nervous system but is also a poison in high enough quantities [2]. Here, we consider uptake to be due to breathing in exogenous CO, and excretion to include both exogenous and endogenous sources. CO is transported across the lungs into the bloodstream and binds preferentially to haemoglobin in the blood, forming carboxyhaemoglobin (COHb); the affinity of haemoglobin for CO is around 210 times greater than that for oxygen [3,4]. Inhaling excess CO can lead to a situation where there is inadequate oxygen transported by haemoglobin, and the human body will then suffer from hypoxia [3,5]. CO poisoning results in symptoms that range from headache to unconsciousness, depending on the dose.

Once exposure to exogenous CO ceases, the body’s mechanisms for excreting CO can return the COHb level to baseline. The typical baseline level of people unexposed to exogenous CO is around 0.8% COHb. For this process, many studies have shown that the half-life for COHb in the body is about 4 h [6].

CO enters the human body through the lungs, is transported via the blood system and enters the tissue/muscle system. Since the CO partial pressure is higher in the vascular system than in tissue, CO enters and can be stored in the tissue/muscle system. This CO transport process is reversible. If the partial pressure of CO is lower in the ambient environment than in the vascular system, then CO is released from the tissue to the blood and then to the lungs to be exhaled [7]. However, due to the stronger affinity of CO for Hb, there is a baseline COHb concentration in the blood. 

Several factors are known to relate to CO uptake and excretion, including minute ventilation rate (V_E_), alveolar ventilation rate (V_A_), arterial oxygen tension, haem mass and haemoglobin mass. V_E_ is the total rate of ventilation, and V_A_ is the rate of the gas exchange via the alveolar surface during normal breathing. There is a relationship between V_E_ and V_A_. The equation used is V_A_ = V_E_ − fV_D_, where f is the respiration rate (1/min), and V_D_ is the dead space (mL) [1,8,9,10].

Haemoglobin is the main oxygen carrier in the human body. It contains a haem prosthetic group that has an iron atom, and it binds to oxygen to form oxyhaemoglobin. By this method, the haemoglobin takes the oxygen through the body [11]. In physiology, CO affects the oxygen–haemoglobin dissociation curve (ODC). Because CO has such a high affinity with haemoglobin, it decreases the blood oxygen concentration significantly [12].

Although several factors relating to CO uptake and elimination in the human body have been described, we did not find an overview of the situation worldwide. This review aimed to summarise the literature on factors that relate to CO uptake and elimination in the human body. Furthermore, we divided the factors into different dimensions to present a clear relationship between each factor. If we understand the factors that affect the rate of CO uptake and elimination, we will be able to predict the CO concentration in the human body and may be able to give suggestions for more effective treatment of CO poisoning.

In this paper, several factors are described that relate to the rate of CO uptake and elimination, which include environmental, demographic, physiological and treatment factors. The related factors contain different dimensions, from physical exposure to physiological metabolism.

## 2. Materials and Methods

### 2.1. Scope and Search Strategy

Scientific databases, including PubMed, EMBASE and Web of Science, were searched for studies. The search strategy used a combination of keywords related to carbon monoxide poisoning and elimination, carbon monoxide poisoning and uptake, carbon monoxide poisoning and half-life and carbon monoxide poisoning and equation. We also manually searched the references of every primary study and review article for further publications to make sure all relevant publications were included.

### 2.2. Inclusion and Exclusion Criteria

In the literature review, certain study inclusion and exclusion criteria were applied. Particularly, the review only included data-based studies on human subjects that appeared in peer-reviewed journals in the English language that had the full text available. Theses, dissertations or presentation abstracts that were not published in peer-reviewed journals were excluded. Also, the authors screened the titles and abstracts to exclude irrelevant publications.

### 2.3. Search Results and Study Characteristics

**Figure 1:**
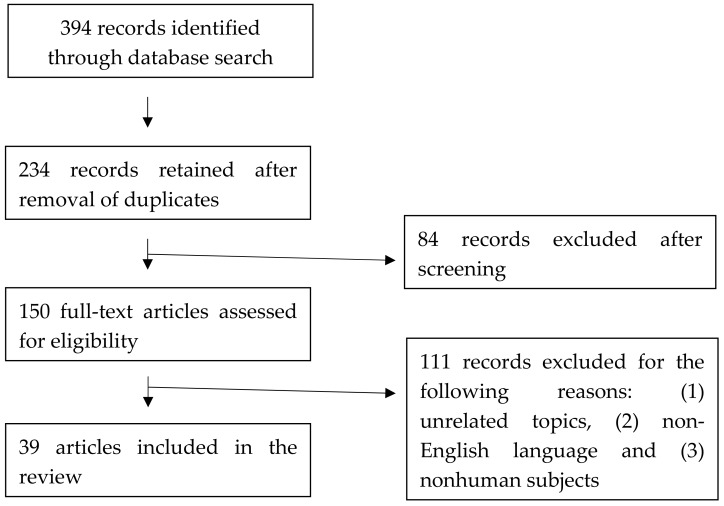
Summary of review process.

The initial search identified 394 studies by the keywords. The references from the papers were checked to see if there were papers that needed to be considered. After deleting duplicative papers and screening the titles and abstracts, 39 studies met the criteria (Figure 1). We identified 39 studies published since 1945 and the search was completed on 16 December 2019.

## 3. Results and Discussion

These 39 studies were divided into four aspects, including environmental, demographic, physiological and treatment factors. 

### 3.1. Environmental Factors

When measuring the rate of CO uptake and elimination, the first point to consider is the dose of CO to which the subjects are exposed. There are several environmental factors related to CO exposure, including CO concentration in the ambient air, the duration of CO exposure, the oxygen concentration in the ambient air and altitude.

#### 3.1.1. CO Concentration in Ambient Air and Duration

From the literature, the main factor that may relate to the rate of CO uptake and elimination is ambient CO concentration. In Forbes et al.’s study, the authors obtained more than 100 observations from seven healthy male laboratory workers. When the concentration of CO increased in the inspired air, the rate of CO uptake would also rise [13]. Moreover, Peterson and Stewart created an experiment for 22 subjects. Two subjects (subjects 21 and 32) breathed in 200 ppm CO, and two other subjects (subjects 1 and 12) breathed in 100 ppm CO. As a result, for subjects who breathed in 200 ppm CO, their COHb reached 10% in around 60 min. However, for the subjects who breathed in 100 ppm CO, their COHb reached 10% by 200 min later [8].

The duration of CO exposure affects CO uptake and elimination. In a multicompartment model, researchers tried to predict the CO washout time from different durations. Bruce and Bruce matched the simulation model with the measured data from Benignus et al.’s study and found that the model predicted COHb concentration well [14,15]. They simulated the same dose of CO through two different scenarios of CO exposure. One was exposed to 10,000 ppm CO for 5 min, and the other was exposed to 1250 ppm CO for 40 min. The result for the elimination time of the long duration was slower than for the short duration [15].

#### 3.1.2. Oxygen Concentration in Ambient Air

In Forbes et al.’s study, the authors made subjects breathe CO in the air environment and also in a pure oxygen environment. Then, they compared the rate of CO uptake of the subjects. The ratio of CO uptake rate in the pure oxygen environment compared with in air was around 0.77 during rest and 0.62 during hard work. The reason is that there would be much more oxygen competing with CO if the subjects breathe CO in oxygen than in air. Therefore, the CO uptake will also be slower in oxygen than in air [13].

#### 3.1.3. Altitude

Some researchers found that altitude may be a factor that governs the rate of CO uptake and elimination [13,16]. Collier and Goldsmith modified the Coburn–Forster–Kane (CFK) equation by adding altitude as a factor affecting CO uptake and elimination [16]. For example, the partial pressure of oxygen decreases when the altitude increases. Therefore, when people breathe the same amount of CO, it may cause a higher CO concentration at higher altitudes than at sea level. The reason may be due to the lower partial pressure of oxygen at high altitudes, which means there is less oxygen to compete with CO and the COHb half-life increases. Moreover, altitude may also affect the ODC to the left, which increases haemoglobin’s affinity to bind to oxygen [16]. However, Forbes et al. recorded the CO uptake of three subjects at sea level, 16,000 ft and 40,000 ft. The results showed that the CO uptake rate increased by increasing the altitude due to higher V_E_ [13]. 

All the factors described above are mainly divided into three parts, namely, the CO concentration, the duration of CO exposure and the partial pressure of CO. When people are exposed to high concentrations of CO or high partial pressure of CO, the rate of CO uptake increases. However, when considering the duration of CO exposure, even though subjects are exposed to a lower concentration of CO, they have a longer elimination time if the exposure time is increased. Both the CO concentration and duration of CO exposure are critical environmental factors related to the rate of CO uptake by, and washout from, the human body.

### 3.2. Demographic Factors

In many disease-related studies, we could find demographic factors that may be relevant to the disease [17,18]. In some studies, age and sex were reported to relate to the rate of CO uptake and elimination [19,20].

#### 3.2.1. Age

In Klasner et al.’s study, the authors focused on CO poisoning in the paediatric population. Compared with previous studies, they found that children had a shorter COHb half-life than adults. For 26 children, the mean half-life of COHb was 44.0 min on 100% oxygen at 1 atm. However, the half-life of COHb in adults was around 80 min in the same situation. The authors assumed that the reason for this was the difference in minute ventilation between children and adults. Although children have a smaller tidal volume than adults, they have faster respiratory rates, which leads to an increase in their V_E_ [19,21].

Moreover, there are still several factors that may change with age, including the volume of haem, blood volume, muscle myoglobin mass and lung function. Therefore, further studies need to be done to understand the age effects.

#### 3.2.2. Sex

Tracing back to Pace et al.’s study, they found a sex-related difference in the half-life of COHb. The half-life of COHb elimination by breathing 100% oxygen at 2.5 atmosphere absolute (ATA) was 22.3 min for men and 15.1 min for women. However, the authors did not explain the reason for the sex-related difference [22]. Although some studies showed a sex-related difference, there were still other studies that found no sex-related difference in the rate of CO uptake and elimination [9,23]. In a large natural experiment, 184 people were exposed to CO in a public high school for around 2.5 h. The researchers gave questionnaires to the victims and analysed the data. They found no differences between ages, sexes and smokers and nonsmokers [23]. Moreover, Weaver et al. found that sex did not have a significant influence on half-life [9].

Zavorsky et al. did find a sex-related difference for the half-life of CO elimination and revealed the factors behind this effect. The results showed that women had a shorter half-life of CO elimination than men. The factors found to influence the rate of CO elimination were V_A_ and total haemoglobin mass [20].

#### 3.2.3. Smoking

When someone smokes a cigarette, the smoker is likely to be exposed to CO concentrations of around 400–500 ppm and experience a higher COHb concentration than a nonsmoker. The COHb concentration is usually less than 5% in nonsmokers and more than 5% in smokers [24]. Another study also showed that COHb is different between smokers and nonsmokers in London. Smokers have COHb levels of around 5%–8% compared with nonsmokers, who have COHb levels of about 1%–3% [25]. This is in contrast with the study of Burney et al. where no differences were observed [23].

#### 3.2.4. Exercise

The level of exercise or activity of subjects may have some influence on the rate of CO uptake and elimination. In Forbes et al.’s study, the rate of CO uptake in the subjects was higher during hard work than during rest [13]. Filley et al. also found that the rate of CO uptake was different between subjects at rest and exercise. When the subjects increased the level of exercise, the minute ventilation and the rate of CO uptake also rose [26]. However, the rate of CO uptake was not significantly different between either a low (~45 W) or moderate (~90 W) power output measured by a cycle ergometer in an experiment involving 29–37-year-old subjects [27].

Demographic factors, such as age, sex and exercise, are related to the rate of CO uptake and elimination. However, the physiological factors of minute ventilation, alveolar ventilation and total haemoglobin mass likely explain the demographic observations. 

### 3.3. Physiological Factors

When people breathe in CO, the CO gas enters the lungs and then transfers via the alveoli into the vascular system. Through the blood circulation, most of the CO binds to haemoglobin and is transferred from the arterial to the venous blood. Besides the blood, some of the CO also crosses into the tissue and binds to it, leading to the formation of carboxymyoglobin [7]. Consequently, like lung and cardiovascular functions, muscle function may play a role in CO circulation in the human body and it is related to the rate of CO uptake and elimination. In Penney’s book, he stated that the two main physiological factors that affect the rate of CO uptake and elimination are the ventilation and diffusion rates of CO [28].

#### 3.3.1. Lung Function

##### Ventilation Rate

Many studies have discovered that the ventilation rate may affect the rate of CO uptake and elimination [1,13,19,26,29,30]. When people breathe at a high ventilation rate, they tend to absorb more CO into the lungs and blood. However, a high ventilation rate can also exhale more CO than a low ventilation rate over the same duration [1,13]. In a study by Zavorsky et al. (2014), the results showed that men have a more prolonged washout time of CO than women, and the authors tried to explain the result. After they tested different factors in the subjects, they found that the alveolar ventilation and total haemoglobin mass may be the reasons that explain the difference in the CO half-life. When people have increased alveolar ventilation, the CO elimination time decreases [20].

However, in Bruce and Bruce’s model (2006), they found that the half-life of COHb has a higher correlation (r = 0.714) with Vb/VAwo (blood volume/ventilation during washout) than ventilation alone. Because the CO is exhaled directly from the lungs and carried by the blood, the limiting factor may be this ratio [15].

##### Diffusion Capacity of CO (DL_CO_)

Between the alveolar and pulmonary capillaries, gas passes the pulmonary membrane by simple diffusion. The diffusion capacity “is the ability of the lungs to transfer gas from inhaled air to the red blood cells in pulmonary capillaries” [26]. The diffusion capacity is affected by molecular species, body size, rate of work, temperature and pressure [8]. The DL_CO_ is widely used to test patients’ lung function in hospitals nowadays [31]. The mean values for DL_CO_ were found to be 28.05 ± 5.07 mL/min/mmHg for men and 20.79 ± 4.03 mL/min/mmHg for women [32]. The CFK equation, an equation for the study of the endogenous production of CO, CO distribution, CO uptake and elimination, contains the pulmonary diffusing capacity as a factor that may affect the rate of CO uptake and elimination. When the diffusion capacity is higher, it means that CO has a great ability to pass through the membrane, and the rate of the CO uptake and elimination is increased [1,33,34]. However, in Filley et al.’s study, the authors found that the ventilation rate may play a more important role in the rate of CO uptake and elimination [26].

##### Chronic Obstructive Pulmonary Disease (COPD)

COPD is defined as an obstruction of the airways that makes it hard to breathe. COHb levels were found to be significantly higher in COPD patients compared with the normal population [35,36]. Some COPD patients have a lower diffusing capacity for CO in the lungs [37,38,39]. In Crowley et al.’s study, their data suggested that the half-life of COHb is around 6.5 h in COPD patients compared with healthy subjects, who have a COHb half-life of about 2–5 h [40]. Therefore, COPD patients may have a slower rate of CO elimination than healthy people due to the lower gas exchange and poor respiratory mechanics. However, Crowley et al. explained that there was no dramatic difference of COHb half-life between COPD patients and normal subjects, so it might be the sedentary life of COPD patients that causes the longer COHb half-life [40].

#### 3.3.2. Cardiovascular Function

##### Blood Volume

When CO enters the vascular system, most of the CO combines with haemoglobin as COHb. At the end of CO exposure, most of the CO stays in the blood. Consequently, the blood volume may be an important factor that relates to CO uptake and elimination. In Pugh’s study, the average blood volume was around 78 mL/kg [41]. In the CFK equation, blood volume is one of the factors affecting the rate of CO uptake and elimination [1]. Furthermore, in Bruce and Bruce’s study, their model predicted that if people have a large blood volume, they carry more CO in the body and have an increased rate of CO uptake and elimination [15].

##### Haemoglobin Mass

Haemoglobin is the crucial factor that determines the maximum amount of oxygen uptake. The average haemoglobin mass is about 11.6 g/kg [41]. However, when compared with oxygen, CO has around 210 times greater affinity for haemoglobin [4]. In Zavorsky et al.’s study, the authors suggested that the total haemoglobin mass affects the rate of CO uptake and elimination [20]. However, the effects of total haemoglobin mass on the rate of CO uptake and elimination require further investigation.

##### Diffusion Rate of CO Flux from Blood to Muscle Compartment

The blood-to-muscle diffusion coefficient (Dmco) refers to the diffusion rate of the CO entering the muscle compartment. In a multicompartment model, Bruce and Bruce (2006) set the Dmco to zero and tried to determine how it would influence the half-life for CO washout. When the Dmco was set to zero, the half-life increased. Therefore, this means that no CO entered the muscle compartment, and all the CO decreased by exhalation from the lungs [15]. Moreover, in 2008, the authors tested the model with experimental data, including human and animal data [14,23,42,43]. They found their model could fit well with the experimental data when changing the Dmco in different conditions [44].

##### Muscle Mass

Not only can haemoglobin bind to CO in muscle cells, but myoglobin also contains haem, to which CO can bind. Muscle tissue can take up CO over a prolonged period, even after the end of exposure. For a young adult male, the muscle compartment may account for about 41% of the total body mass [45]. In their study, Möller and Sylvén assumed that every gram wet weight of muscle would contain about 4.7 mg of myoglobin [46]. Take a 70 kg man, for example, who may, approximately, have 135 g of myoglobin. Each myoglobin molecule contains a haem molecule that could bind up to 178 mL of CO. Therefore, the muscle compartment could be an essential place to store CO and increase the half-life of CO elimination. Although muscle may be a factor, it is less critical for the half-life of COHb. The reason is that the volume of CO removed from muscle is less than the volume of CO removed from the blood [15].

##### Anaemia

Anaemia refers to a low haemoglobin level or low red blood cell count in the blood or increased destruction of red blood cells. In Woehlck et al.’s study (2001), the authors predicted that patients would have more severe CO poisoning according to the haematocrit level. They explained that patients with low haemoglobin tend to have a higher COHb concentration than people with normal haemoglobin after exposure to CO. When the subjects breathed in the same CO concentration, the rate of COHb increased more rapidly in the subjects with a lower haematocrit level than a higher haematocrit level [47].

Among physiological factors, besides ventilation rate and diffusion capacity (which have been emphasised for a long time), there are still many factors that need to be considered. For example, our review indicates that blood volume, total haemoglobin mass, muscle mass and disease may influence the rate of CO uptake and elimination. However, the physiology of the human body is known to be complicated. Some factors may have a relationship with other factors. Isolating the role of any specific factor will require careful study. 

### 3.4. Treatment Factors

The most common treatment for CO poisoning is breathing 100% oxygen as soon as possible. Moreover, high-pressure oxygen or hyperbaric oxygen (HBO) therapy also has been used in several countries as a solution for CO poisoning. The rate of CO elimination may relate to the atmospheric pressure or percentage of oxygen. Higher atmospheric pressure and percentage of oxygen result in a faster CO elimination rate [19,29].

#### 3.4.1. 100% Oxygen

Weaver et al. (2000) conducted a study to understand which factors may influence the COHb half-life. Through their retrospective chart review from 1985 to 1995, they showed that the half-life of COHb decreases with the increase of arterial oxygen tension. As a result, they found that the half-life of COHb was around 74 min for patients treated with 100% oxygen at atmospheric pressure, which was shorter than for patients only breathing in air [9]. 

There are several methods to provide 100% oxygen to patients, such as a rebreathing reserve mask, high-flow nasal cannula (HFNC) oxygen and oxygen therapy with continuous positive airway pressure (CPAP) [48,49,50,51]. In Kim et al.’s (2019) study, HFNC did not reduce the CO half-life compared with a rebreathing reserve mask [49]. When comparing normobaric oxygen therapy with 1 h of CPAP therapy, Bal et al. discovered that patients receiving CPAP therapy had a shorter CO half-life than those receiving normobaric oxygen therapy. The authors assumed that CPAP therapy increases the gas exchange area and improves ventilation due to the positive pressure going into the alveoli [50].

#### 3.4.2. HBO Therapy

Treating CO-poisoned patients with HBO therapy is still controversial [6,52,53]. There are different policies in different countries. However, Pace et al. found that high-pressure oxygen could increase CO elimination in CO-poisoned patients [22]. In Ernst and Zibrak’s study, they found that the half-life of COHb would be approximately 4 h on air, 1.5 h on oxygen and less than 20 min during HBO therapy [54].

#### 3.4.3. Carbogen

Carbogen is a mixture of carbon dioxide and oxygen gas. Usually, CO_2_ is set at 5%–10% in O_2_ [55,56]. When patients breathe in carbogen, their brain CO_2_ sensor detects that more CO_2_ is stored in the body, and as a result, the brain sends a signal to increase alveolar ventilation, thus decreasing the half-life of COHb [56,57].

The 100% oxygen and HBO therapies are based on the theory that the alveolar partial pressures of oxygen would be affected by the inhaled partial pressure of oxygen. When increasing the partial pressure of oxygen, there is more oxygen that can compete with CO to bind with haemoglobin. Then, the rate of CO elimination would be raised. However, using HBO therapy for CO-poisoned patients is still controversial. Although HBO therapy is not recommended for CO-poisoned patients in the United Kingdom, it is a treatment for CO-poisoned patients that is widely used in Taiwan.

## 4. Conclusions

In the literature review, some environmental, demographic, physiological and treatment factors were found to have an impact on the rate of CO uptake and elimination (Table 1 and Table 2). Among environmental factors, the rate of CO uptake increases by raising the CO concentration or reducing the oxygen concentration of the inhaled gas. Moreover, the altitude can alter the rate of CO uptake due to the different partial pressure of oxygen at different altitudes. The duration of CO exposure is an important factor. If people were exposed to CO for a long time, even if the concentration of CO were low, it would also have an adverse impact and reduce the rate of CO elimination. That is why attention is needed not only for acute CO poisoning but also chronic CO poisoning, which is often ignored.

Demographic factors, such as age, sex, smoking and exercise, are not direct factors relating to the rate of CO uptake and elimination. Included in these factors may be physiological factors, such as minute ventilation, alveolar ventilation and total haemoglobin mass, which are direct factors affecting the rate of CO uptake and elimination. Other physiological factors, including muscle mass and diffusion capacity, can also influence the rate of CO uptake and elimination. Consider the treatments, for example: the three treatments for CO poisoning increase the rate of CO elimination by raising the partial pressure of oxygen and the ventilation rate. Therefore, these treatments are based on the physiological reactions that have been tested in past studies.

This review discussed most of the factors that impact the rate of CO uptake and elimination. Information remains limited and there are numerous other potentially important factors that could influence CO update and elimination, such as genetics, disease, vulnerable groups, children, the elderly, weight and so forth. Thus, there may be different treatment strategies for groups with different characteristics. Further studies focused on this field may find better ways to increase the rate of CO elimination in CO-poisoned patients. 

## Figures and Tables

**Table 1:** The factors related to CO uptake.

Field	Factor	Results	Experiment	Control	Reference
Environment	CO concentration increase	CO uptake rate increase	Range: 0.01%–0.2% CO		Forbes et al. (1945)
			Range: 0–523 CO ppm		Peterson and Stewart (1970)
			Range: 8.7–1000 CO ppm		Peterson and Stewart (1975)
	Duration of exposure longer	CO uptake amount increase	Range: 0–270 min		Forbes et al. (1945)
			Range: 15–480 min		Peterson and Stewart (1970)
			Range: 0–1440 min (50 CO ppm)		Benignus et al. (1994)
	O_2_ concentration increase	CO uptake rate decrease	Oxygen	Air	Forbes et al. (1945)
	Altitude increase	CO uptake rate increase	16,000 ft; 40,000 ft	0 ft	Forbes et al. (1945)
	Exercise increase	CO uptake rate increase	Hard work	Rest	Forbes et al. (1945)
		CO uptake rate increase	Light exercise; moderate exercise	Resting	Filley et al. (1954)
		No difference	Moderate exercise	Low exercise	Tikuisis et al. (1992)
Physiology	Ventilation rate increase	CO uptake rate increase	Range: 6–30 L/min		Forbes et al. (1945)
		CO uptake rate increase	Range: 5.8–105 L/min		Filley et al. (1954)
	Diffusion capacity of CO (DL_CO_) increase	CO uptake rate increase	36.3 cm^3^/min/mmHg	16.9 cm^3^/min/mmHg	Filley et al. (1954)
		CO uptake rate increase	Range: 5–30 mL/min/torr		Bruce and Bruce (2003)
		CO uptake rate increase	-	-	Gosselin et al. (2009)
	Blood volume increase	CO uptake rate increase	-	-	Coburn et al. (1965)
	Diffusion rate of CO flux from blood to muscle compartment in crease	CO uptake rate increase	Range: 0–100 mL/min/torr		Bruce et al. (2008)
	Muscle mass	Less important	-	-	Bruce and Bruce (2006)
	Anaemia	CO uptake rate increase	Haematocrits of 18% and 30%	Haematocrits of 42% and 60%	Woehlck et al. (2001)

Note: 1 torr = 1 mmHg, a unit of pressure based on an absolute scale; 1 cm^3^ = 1 mL.

**Table 2:** The factors related to CO elimination.

Field	Factor	Results	Experiment	Control	Reference
Environment	CO concentration increase	CO half-life longer	200.8 CO ppm for 60 min	51.6 CO ppm for 60 min	Peterson and Stewart (1970)
	Duration of exposure longer	CO half-life longer	1250 CO ppm for 40 min	10,000 CO ppm for 5 min	Bruce and Bruce (2006)
			(same CO dose in two groups)		
	O2 concentration increase	CO half-life shorter	100% oxygen	-	Weaver et al. (2000)
			2.5 atm, 100% oxygen (HBO)	-	Pace et al. (1950)
Demography	Age increase	No difference	Range: 9–86 years old		Burney et al. (1982)
			>40 years old	<40 years old	Weaver et al. (2000)
		CO half-life shorter	4–12 years old	-	Klasner et al. (1998)
	Sex	No difference	Female	Male	Burney et al. (1982)
			Female	Male	Weaver et al. (2000)
		CO half-life shorter	Female	Male	Pace et al. (1950)
			Female	Male;	Zavorsky et al. (2014)
	Smoking	No difference	Smokers	Nonsmokers	Burney et al. (1982)
Physiology	Ventilation rate increase	CO half-life shorter	Range: 4–10 L/min		Coburn et al. (1965)
			15 and 30 L/min	3 and 6 L/min	Selvakumar et al. (1993)
			Range: 5–20 L/min		Kreck et al. (2001)
			Range: 4–40 L/min		Zavorsky et al. (2014)
	Chronic obstructive pulmonary disease (COPD)	No difference/CO half-life slightly longer	COPD patients	Normal subjects	Crowley et al. (1989)
	Blood volume increase	CO half-life shorter	-	-	Coburn et al. (1965)
			Range: 0.3–0.7 (Vb/VAwo)		Bruce and Bruce (2006)
	Haemoglobin mass increase	CO half-life longer	Male	Female	Zavorsky et al. (2014)
	Diffusion rate of CO flux from blood to muscle compartment increase	CO half-life shorter	Range: 0–2 mL/min/torr		Bruce et al. (2003)
	Muscle mass	Less important	-	-	Bruce and Bruce (2006)
	Anaemia	CO half-life shorter	Anaemia	Polycythaemia	Zavorsky et al. (2014)
Treatment	100% oxygen	CO half-life shorter	100% oxygen	-	Weaver et al. (2000)
	High-flow nasal cannula (HFNC)	No difference	100% oxygenwith high flow	100% oxygen	Kim et al. (2019)
	Continuous positive airway pressure (CPAP)	CO half-life shorter	100% oxygenwith positive pressure	100% oxygen	Bal et al. (2019)Caglar et al. (2019)
	Hyperbaric oxygen (HBO) therapy	CO half-life shorter	2.5 atm, 100% oxygen	-	Pace et al. (1950)
			3 atmosphere absolute (ATA), 100% oxygen	1 ATA, 100% oxygen	Peterson and Stewart (1970)
	Carbogen	CO half-life shorter	Hyperventilation(6% CO_2_ in O_2_)	Without isocapnia	Sein Anand et al. (2017)

Note: 1 torr = 1 mmHg, a unit of pressure based on an absolute scale.
